# Reproductive Endocrine Disorders: A Comprehensive Guide to the Diagnosis and Management of Infertility, Polycystic Ovary Syndrome, and Endometriosis

**DOI:** 10.7759/cureus.78222

**Published:** 2025-01-30

**Authors:** Samra Saleem Azam, Sheetha Vasudevan, Warda Saqib Bukhari, Jainisha Thadhani, Hafsa Tasneem, Shreya Singh, Ijeoma Chijioke, Bruna Mendes de Freitas, Maleesha Bhagyani Weerasinghe Thammitage, Jatin Motwani

**Affiliations:** 1 Department of Medicine, Dow Medical College, Karachi, PAK; 2 Department of Obstetrics and Gynecology, Avalon University School of Medicine, Willemstad, CUW; 3 Department of Obstetrics and Gynecology, Islam Medical and Dental College, Sialkot, PAK; 4 Department of Medicine, Royal College of Surgeons - Medical University of Bahrain, Muharraq, BHR; 5 Department of Obstetrics and Gynecology, Shri Atal Bihari Vajpayee Medical College and Research Institute, Bangalore, IND; 6 Department of Obstetrics and Gynecology, Ivan Horbachevsky Ternopil National Medical University, Ternopil, UKR; 7 Internal Medicine, Ross University School of Medicine, Bridgetown, BRB; 8 Department of Internal Medicine, State University of Pará, Belem, BRA; 9 Department of Family Health, Aloka Health Care, Horana, LKA; 10 Department of Medicine, Liaquat National Hospital and Medical College, Karachi, PAK

**Keywords:** diagnosis, endometriosis, infertility, polycystic ovary syndrome, reproductive endocrine disorders

## Abstract

Reproductive endocrine disorders, including infertility, polycystic ovary syndrome (PCOS), and endometriosis, significantly impact women’s reproductive health and overall well-being. This comprehensive review explores the diagnosis and management strategies for these prevalent conditions. Infertility, affecting millions globally, is often linked to ovulatory dysfunction, PCOS, and endometriosis. PCOS is characterized by hyperandrogenism, menstrual irregularities, and insulin resistance, contributing to anovulation and infertility. The Rotterdam criteria are widely used for PCOS diagnosis, and management includes lifestyle modifications, pharmacological treatments like ovulation inducers, and, in some cases, surgical interventions. Endometriosis, caused by the presence of endometrial-like tissue outside the uterus, leads to chronic pain and infertility through inflammation, adhesions, and impaired ovarian function. Laparoscopy remains the gold standard for diagnosing endometriosis, and treatment focuses on pain relief, fertility preservation, and reducing recurrence. In cases of endometriosis-related infertility, assisted reproductive technologies (ARTs) like in vitro fertilization (IVF) are often recommended. In addition, the role of diet and lifestyle changes in managing these conditions is gaining recognition. This review emphasizes the complexity of reproductive endocrine disorders and underscores the need for individualized treatment plans, combining medical, surgical, and lifestyle approaches to improve fertility outcomes and enhance the quality of life for affected women. The review also highlights the importance of early diagnosis and advances in therapeutic interventions to ensure optimal patient care in the management of infertility, PCOS, and endometriosis.

## Introduction and background

Reproductive endocrine disorders comprise a variety of conditions, including infertility, polycystic ovary syndrome (PCOS), and endometriosis. In addition to having a great impact on physical health, these disorders also have significant psychological, social, and economic consequences, which include anxiety, sadness, depression, and relationship conflicts [1]. Due to the major effects on the reproductive health and overall well-being of afflicted persons, healthcare practitioners must have a comprehensive understanding of the diagnosis and management of the above disorders.

Infertility is a serious global health problem that affects around 50 to 70 million couples across the world [2]. According to the World Health Organization infertility is the inability to conceive even after having regular sexual intercourse for 12 or more months without the use of any contraception [3]. In women, the most common causes of infertility are ovarian dysfunction with anovulation, PCOS, endometriosis, pathology related to the oviducts or uterus, fibroids, and adhesions. Particularly, endocrine disorders like PCOS and endometriosis leading to oligo/anovulation play an important role in female infertility. Tests or procedures essential for the diagnosis of infertility in females include antral follicular count, hormonal assays, hysterosalpingogram, diagnostic hysteroscopy, and diagnostic laparoscopy, which are necessary to determine the underlying cause and to plan the appropriate management [2].

PCOS is one of the most prevalent reproductive endocrine disorders in women [4,5]. Approximately 8% to 13% of women who are of reproductive age suffer with PCOS [6]. The primary manifestations of PCOS are hyperandrogenism and ovulatory dysfunction. Other symptoms include hirsutism, acne, and menstrual irregularities. PCOS is a multifactorial syndrome and its development is also influenced by genetic and environmental factors, which include a sedentary lifestyle, obesity, and consumption of food high in carbohydrates and fats [7]. PCOS causes metabolic abnormalities such as insulin resistance, a greater risk of developing type 2 diabetes, and other cardiovascular diseases [4]. Due to the abnormal proliferation of follicles, PCOS can lead to anovulation and low oocyte quality, which eventually results in infertility [7]. The Rotterdam Criteria provides the basis for the diagnosis of PCOS, which includes ovulatory dysfunction such as oligoovulation/anovulation, clinical and/or biochemical features of hyperandrogenism, and polycystic ovarian morphology in ultrasound [5]. Management of PCOS includes lifestyle modifications such as dietary changes and engaging in physical activity to reduce weight and pharmacological treatments like combined oral contraceptive pills to decrease androgen levels and normalize the menstrual cycles. Sometimes surgical procedures are performed to improve ovarian function and fertility outcomes [8]. For obese women with PCOS, bariatric surgery may be a helpful way to achieve fertility and increase the likelihood of pregnancy [9].

Endometriosis is an inflammatory and chronic disease in which tissue similar to the endometrium is present outside the uterine cavity. Endometriosis affects nearly 10% of women during their reproductive years [10]. The various clinical and molecular evidence put forward various theories regarding the pathogenesis of endometriosis: these include the theories of retrograde menstruation, immunological dysfunction, benign metastasis, metaplasia of coelomic lining, hormonal imbalances, and stem cell and epigenetic regulatory alterations [11]. It is the most prevalent cause of chronic pelvic pain in women and is linked with a markedly higher incidence of infertility compared with women without endometriosis [10,11]. Infertility due to endometriosis is mainly caused by inflammation, altered pelvic architecture, adhesions, impaired ovarian function, and decreased endometrial receptivity. Since the diagnosis can be confirmed by surgical findings, a transvaginal sonogram based on the IDEA consensus can help in diagnosing and staging the disease non-invasively [12]. The objectives of medical treatment for endometriosis include pain management, enhancing the quality of life, preserving fertility, and preventing disease recurrence [11,13]. Consequently, in vitro fertilization (IVF) can prove to be helpful in women with moderate to severe, as well as recurring endometriosis [11].

At present, a single, effective diagnostic method or treatment plan for reproductive endocrine disorders does not exist. This narrative literature review aims to provide a comprehensive overview of the current diagnostic procedures and management strategies for infertility, PCOS, and endometriosis. This review tries to highlight the potential therapeutic pathways that are currently being explored to improve patient care and fertility outcomes for women suffering from reproductive endocrine disorders.

## Review

Pathophysiology

Infertility is a complex illness caused by various physiological and pathological factors. Pathophysiology frequently involves interactions among endocrine, inflammatory, and anatomical variables. Some common causes include ovulation disorders, congestion or blockage in the fallopian tubes, and endometriosis [14]. Polycystic ovary disorder (PCOS), a condition that modifies hormone levels and causes abnormal ovulation or anovulation, can also alter hormonal cycles required for conception and cause problems with fertility [15]. Conditions that include irritation, such as pelvic inflammatory disease (PID), can also result in fertility issues. PID is commonly caused by a lack of treatment for infections contracted through sexual contact, such as chlamydia trachomatis, and leads to fallopian tube injury [14,16,17]. This injury may cause the tubes to end up blocked and halt sperm cells from moving through the tubes to reach an egg or stop a fertilized egg from entering the uterus, causing an increase in the chances of ectopic pregnancy and infertility [14]. In addition, infertility can be caused by endometriosis, where tissue from the endometrium is found external to the uterus, in some cases seen growing on ovaries, within the fallopian tubes, or on other structures within the pelvic cavity [6,18]. This tissue develops similarly to endometrium when hormones are altered within the menstrual cycle, can become inflamed and agonizing, and may cause tissues to create scars or adhere together [19]. As a result, pelvic structures can become irregular, and this may influence the activities of ovulation and fertilization and conjointly affect the chances of a fetus getting embedded in the uterus lining [14,19]. The alterations within the balance of hormones, including those coming from the thyroid and the pituitary organ, can affect the levels of necessary hormones, such as follicle-stimulating hormone (FSH) and luteinizing hormone (LH), that are needed for propagation and expected ovulatory cycles. If the levels of these hormones become anomalous, this may result in menstrual cycles being sporadic, disturbed ovulation, and ultimately infertility [19,20]. 

The pathophysiology of PCOS is complex and controlled by many variables, which include hereditary and hormonal components, as well as factors in a person’s surroundings all working as a composite and combining to affect the well-being of a woman. The syndrome is caused by an abnormal increase in androgens, flawed ovulatory activity, and ovaries with numerous cystic structures. PCOS is mainly driven by an abundance of androgen hormones, and this hormonal imbalance has a great effect on the expression of the disorder. When the ovaries produce an abnormally increased number of hormones, follicle growth is disturbed, and this prevents the advancement of ovulatory follicles and drives numerous anovulatory cycles. This condition is often exacerbated by insulin resistance and is seen in more than half of women suffering from PCOS. An increase in levels of insulin also causes an increase in the making of androgen hormones from the ovaries but causes a decline in the secretion of sex steroid-binding globulin and greater amounts of unbound androgen [21,22]. The pathogenesis of PCOS also includes inflammation and injury from free radicals that add to the disease process. Inflammation that remains over time also affects insulin resistance and increased production of androgens, causing a persistent cycle that maintains the manifestations of PCOS [15].

**Figure 1:**
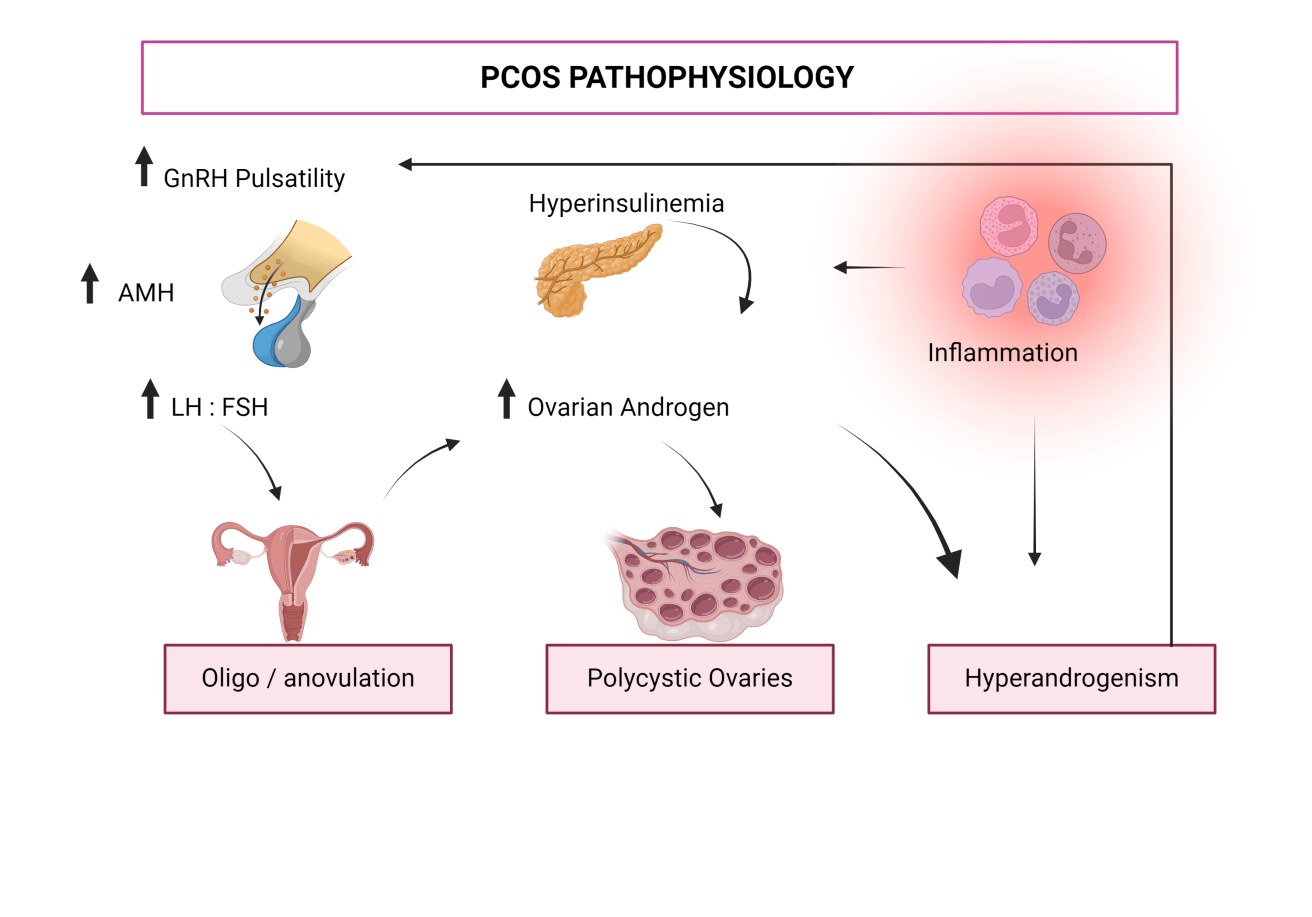
Pathophysiology of polycystic ovary syndrome (PCOS) Created in BioRender (Mendes De Freitas B (2024), BioRender.com/a42y313)

 Figure 1 illustrates the complex interplay of factors contributing to PCOS.

Although there is no consensus on the main cause of this disease, some ideas have been put forward. The most prominent theory is that of retrograde menstruation, according to which the portions of endometrium that had sloughed off may migrate in a retrograde manner out of the uterus through the fallopian tubes, implant on surrounding tissues and peritoneal surfaces, and eventually invade pelvic structures [6,18]. Another well-known explanation for the development of endometriotic lesions is the theory of Müllerian remnants, which suggests that endometriosis may be seen in fetuses and children before menarche and is likely caused by abnormal division and deposition of Müllerian tissue while the organs of a fetus are being formed. It is also thought that tissues that develop from the same origins as the uterus, like the ovaries and some parts of the colon, may experience transformation to endometrial tissue and lead to endometriosis. An additional idea suggests that blood cells and stem cells with origins in bone marrow can develop into endometrial tissue in areas outside the uterus, including the peritoneal cavity and other regions such as the lungs [12]. Endometriotic lesions cause surrounding inflammation and are sustained by their invasion of tissues and the imbalance of steroid hormones as well as the hormones estrogen and progesterone. A recent discovery showed overexpression of the antiapoptotic BCL-2 gene in ectopic tissue, which further encouraged the growth of endometrial cells [23]. 

**Figure 2:**
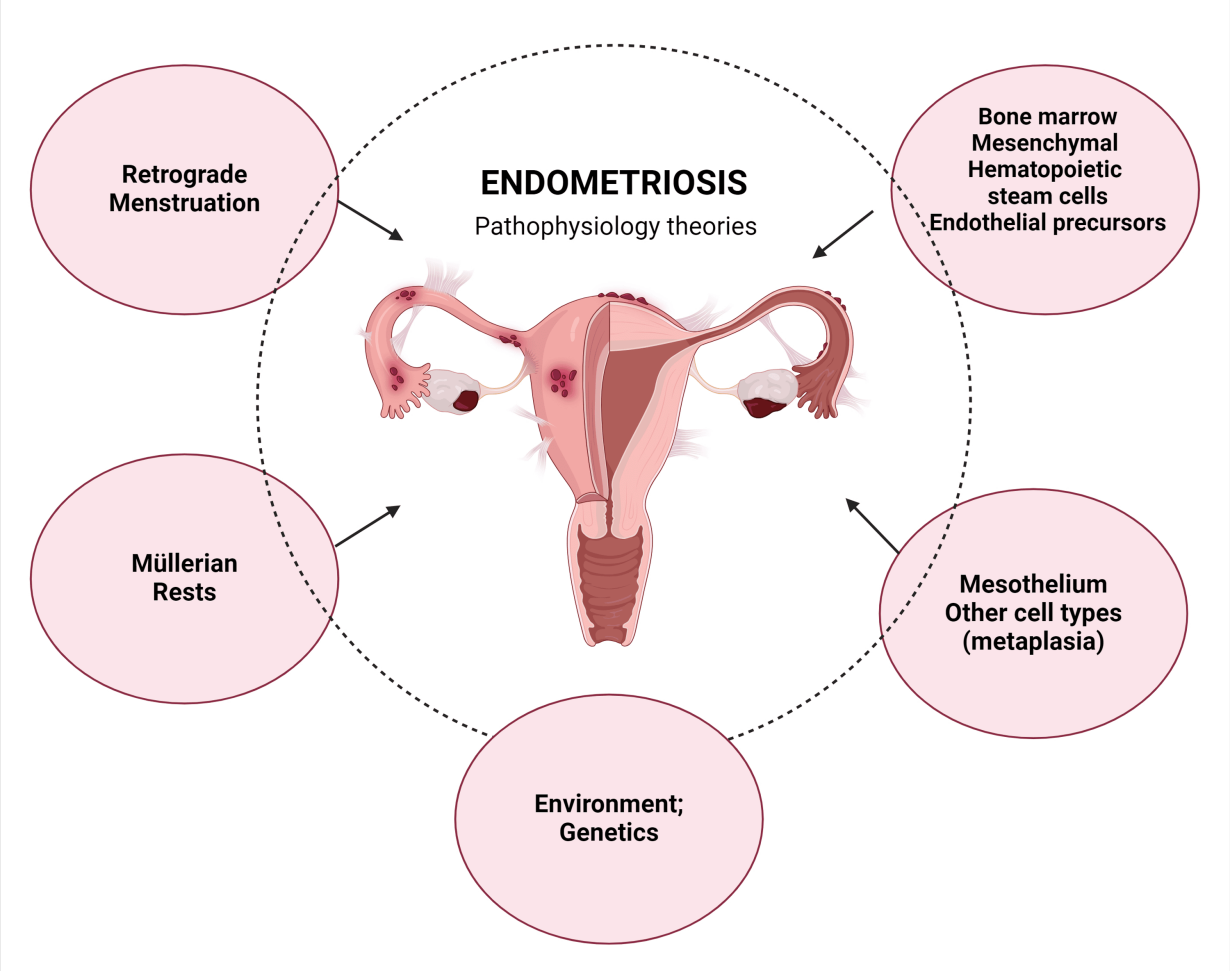
Theories of endometriosis pathophysiology Created in BioRender (Mendes De Freitas B (2024), BioRender.com/q31s807)

Figure 2 outlines the various theories proposed to explain the development of endometriosis.

Diagnosis

A heterosexual woman below 35 years failing to achieve pregnancy after 12 months of regular unprotected sexual intercourse and donor insemination (after six months if they are over 35 years old) should be provided with an evaluation for infertility [24]. It is also important to note that the male partner is also subjected to evaluation simultaneously, as the male factor contributes to approximately 20-30% of cases individually and about 50% of overall infertility cases [25,26]. An infertility evaluation for a woman usually includes the following: 1) in-depth history taking, which must include menstrual history, family history (to rule out genetic causes, venous thrombotic disorders), social and lifestyle history (physical exercise, cigarettes, and illicit drug abuse), sexual history, and a thorough review of systems [27,28]; 2) a physical exam, which involves assessment of BMI, evaluation of thyroid and breast exams, any signs indicating androgen excess, abnormalities in vaginal, cervical, and uterine anatomy, pelvic masses, and transvaginal ultrasonography, commonly done as an initial part of the procedure at the bedside [27,28].

After adequately considering all the patient information, further clinical assessments like specific laboratory and imaging tests are done to confirm the etiology and diagnosis [27,28].

Infertility has multiple etiologies and affects 8-12% of perimenopausal women. Anovulatory infertility is the most common cause, among which 80% have been identified with PCOS. Diagnosing PCOS has been challenging because of widely varying manifestations observed across diverse populations and impacted by multiple factors such as obesity and age of the women with this condition [28].

The diagnosis for PCOS that is widely accepted is based on the Rotterdam criteria, which include the following:

Oligo Ovulation and/or Anovulation

According to Azziz et al., around 75% of women with PCOS have clinically observable menstrual dysfunction, and recent data suggests close to 20% will show apparent eumenorrhea, making it a very important factor to consider during evaluation. Data regarding menstrual irregularities must be promptly collected during comprehensive history-taking. Cycles more than 35 days apart or annually less than eight menses raise suspicions of PCOS [5,29]. 

Clinical and/or Biochemical Signs of Hyperandrogenism

Clinical features of hyperandrogenism mostly seen in PCOS patients are acne, hirsutism, and androgenic alopecia [30]. The modified Ferriman-Gallwey (MFG) method, whose scores range from 0 to 4, is given to the amount of terminal hair growth in nine different body regions: upper lip, chin, chest, upper and lower abdomen, thighs, upper and lower back, and upper arms. A score of 8 or higher indicates hirsutism. MFG scoring is the gold standard method for diagnosing hirsutism. While clinical signs may be helpful in diagnosing PCOS in young adults, these symptoms that normally occur during the pubertal period make it harder to differentiate in adolescents. Hence, biochemical tests such as lab values of free or total testosterone, androstenedione, and dehydroepiandrosterone sulfate (DHEAS) detected using high-quality assays and tests involving radioimmunoassays and electrochemiluminescence are used for better analysis among them [5,30,31].

Polycystic Ovaries

Generally, transvaginal ultrasonography detects enlarged ovaries with cyst-like-looking morphology. More than or equal to 20 follicles in at least one ovary measuring 2-9 mm or either ovary having a volume of more than 10 cm^3^ [32].

A woman meeting two of the three features while excluding other androgen excess or related disorders is formally diagnosed with PCOS. It is important to remember that PCOS is a diagnosis of exclusion [33].

Another leading cause of infertility is endometriosis. The diagnosis of endometriosis may involve the following:

History Taking and Physical Exam

As mentioned above the first and foremost part of the evaluation is the history taking and physical exam. Endometriosis is often associated with heavy menstrual bleeding, dysmenorrhea (cyclic pain), and chronic pelvic pain. Dysmenorrhea and cyclic pain should not be dismissed by the physician in general, and further assessment of the patient is necessary and will help with the early detection of conditions like endometriosis [34]. A physical exam involves the use of a speculum and a bimanual examination. These exams are rarely helpful. In Nehzat et al., physical exams showed a substandard negative predictive value of the pelvic exam. Forty-seven percent of patients who were surgically diagnosed with endometriosis did not show any abnormalities in the bimanual examinations [35]. 

Imaging

Transvaginal ultrasound according to the IDEA (International Deep Endometriosis Analysis group) consensus is recommended as the initial diagnostic approach for patients suspected of having endometriosis [36]. It is one of the most cost-effective imaging modalities and has high specificity and sensitivity in the diagnosis of ovarian endometriosis. Compared to ultrasound, MRI is more accurate and superior with the ability to detect intermediate pelvic masses and rectosigmoid lesions. It is important to note that a negative finding on imaging does not rule out endometriosis, especially superficial peritoneal disease. Diagnosing endometriosis via nonsurgical methods has shortened the mean time from first consultation to diagnosis compared to diagnosis via surgical methods [37]. 

Laparoscopy

It should be considered in cases where imaging remains inconclusive or medical management is unsuccessful. Laparoscopy would show various forms of endometriosis, including peritoneal implants, peritoneal windows, endometriomas, and deep nodules, often with associated adhesions. Laparoscopy visualizes endometriomas that are often associated with ovarian adhesions. Inspection and biopsy of the cyst wall surfaces are important to exclude ovarian neoplasm. Laparoscopic findings are verified through histological analysis although negative histology does not completely rule out the disease [34-35,37].

Biomarkers

Researchers are actively investigating serum markers such as CA125, CA19-9, and leptin for their potential use in diagnosing endometriosis, assessing disease activity, and monitoring treatment efficacy. Several studies have indicated micro RNAs (miRNAs) as a diagnostic marker for endometriosis [38]. However, challenges with peritoneal markers due to hormonal fluctuations and changes in peritoneal fluid levels complicate efforts for standardization. Individual serum markers lack specificity for diagnosing endometriosis or correlating with symptoms, persuading studies to use multiple markers or panels to improve diagnostic accuracy [39]. 

Further research is required to enhance diagnostic strategies, as there are still a lot of shortcomings in the existing ones to ensure that patients receive optimal care. It is also necessary to stress the importance of early diagnosis which would help with proficient management with better outcomes.

Management

Diet and Lifestyle Modifications for PCOS, Endometriosis, and Infertility

Lifestyle modifications are known to improve fertility outcomes in PCOS [40,41]. A study by He et al. highlighted that PCOS with metabolic syndrome negatively impacted female fertility and adversely affected outcomes of in vitro fertilization (IVF). They found that 27.2% of infertile women with PCOS had metabolic syndrome, and these women had longer periods of infertility compared to those without metabolic syndrome (4.0 ± 2.2 years vs. 3.7 ± 2.2 years, p = 0.004) [42]. According to a systematic review by Shang et al., better fertility outcomes were seen in patients with PCOS when introduced to dietary interventions (relative risk (RR) = 2.87, 95% confidence interval (CI): 1.99-4.13; p < 0.00001), without between-grouped heterogeneity (I^2^ = 0%), and improved ovulation rates were seen in the diet groups with minimal treatment (RR = 1.30, 95% CI: 1.10-1.53; p = 0.002; I^2 ^= 0%) [43]. 

In the case of endometriosis, diet may help manage concurrent conditions such as irritable bowel syndrome and painful bladder syndrome. However, its full effects on fertility and endometriosis outcomes are still under investigation [44]. An ongoing study and proposed protocol by Mikocka-Walus et al., comparing yoga and cognitive-behavioral therapy (CBT), aims to assess improvements in quality of life and healthcare costs in women with chronic pain due to endometriosis. CBT sessions can be conducted across an eight-week period alongside pre-prescribed medication for endometriosis, with another group receiving weekly yoga sessions for the same time period. The control group receives educational emails on a weekly basis concerning pain management. Lifestyle management of endometriosis is crucial, as it is a chronically disabling condition that is known to reduce quality of life. Quality of life can be measured using the European Quality of Life Five Dimension survey (EQ-5D-5L) and the endometriosis health profile survey for all groups. Sleep quality should also be investigated using the Jenkins Sleep Scale. Depression, anxiety, fatigue, menstrual symptoms, and pain should also be taken into consideration [45]. IVF is a well-known assisted reproductive technology (ART). Koumparou et al. conducted a pilot RCT to assess the impact of an eight-week stress-management protocol on women undergoing IVF. While the intervention significantly reduced stress (p < 0.001), the study concluded that stress reduction alone did not conclusively improve IVF success rates due to other factors such as participant age and spousal history of cryptorchidism [46]. 

Through a systematic review and meta-analysis, Xie F et al. tried to establish the association between physical activity (PA) and infertility. PA levels were categorized to assess the impact on infertility risk and high PA was defined as engaging in moderate to vigorous physical activity for at least 150 minutes per week, aligning with international guidelines. Low PA refers to activity levels below this threshold. This meta-analysis revealed that individuals with high PA had a significantly reduced risk of infertility, with an RR of 0.58 (95% CI: 0.45-0.74), indicating a 42% lower risk compared to those with low PA. This finding underscores the protective effect of adhering to recommended PA levels against infertility [47].

Medical Management of PCOS, Endometriosis, and Infertility

Ovulation induction is another important step in managing infertility caused by PCOS. This intervention can induce ovulation or induce multiple mature ovarian follicles as a part of an ART or for timed intercourse [48]. Clomiphene citrate (CC), a selective estrogen receptor modifier that promotes the growth of ovarian follicles, and letrozole (LE), an aromatase blocker, are the two main ovulation inducers [48,49]. Wang et al. compared endometrial receptivity using ultrasonic parameters and biomarkers (integrin αvβ3 and VEGF) during the implantation window among PCOS patients undergoing ovulation induction with either LE or CC and those in a natural cycle. Despite no significant difference in ovulation rates between the LE and CC groups, the LE group showed significantly higher ultrasonic parameters and biomarkers compared to the CC and natural cycle groups (p < 0.05). In addition, LE showed higher clinical and ongoing pregnancy rates than the CC group (25.6% vs. 13.3%, 23.3% vs. 11.1%) (p < 0.05), suggesting that LE is more effective for ovulation induction in PCOS [49].

In 2019, Shi et al. compared the effects of LE and human menopausal gonadotropin (HMG) in treating CC-resistant PCOS. After randomizing into two groups, they assessed the response based on the number of growing and mature follicles, serum estradiol (E2), endometrial thickness (ET), pregnancy occurrence, and miscarriage rates. They found no significant difference between the LE and HMG groups for ovulation (53.6% vs. 64.7%, p > 0.05) and pregnancy rates (22.9% vs. 27.1%, p > 0.05). However, the LE group had a lower incidence of ovarian hyperstimulation syndrome (2% compared to 12.5% in the HMG group, p < 0.05), concluding that letrozole-induced ovulation can result in an ovulation rate and pregnancy rate like gonadotropin but reduce the risk associated with treatment. LE is effective and safer for ovulation induction in patients with CC-resistant PCOS [50]. Apart from LE and CC, metformin is also considered an ovulation induction agent in PCOS. Sharpe et al. evaluated the effectiveness and safety of metformin in improving reproductive outcomes for PCOS patients undergoing ovulation induction. With the available data, they concluded that metformin is beneficial over placebo for live births (OR = 1.59, 95% CI 1.00-2.51; I^2^ = 0%; four studies, 435 women; low-quality evidence); however, in the placebo intervention, the risk of gastrointestinal side effects was reported at 10%, whereas with metformin this risk was between 22% and 40%. Data looking at the combination of metformin and CC versus CC alone and metformin compared to CC for live births were inconclusive [51].

A systematic review by Melin et al. established that the combination of metformin and the oral contraceptive pill (COCP) significantly enhanced insulin resistance, in addition to symptoms of excess androgens, compared to just taking the contraceptive pill alone. However, metformin was found to be less effective than COCP in improving free androgen index (FAI) outcomes (mean difference (MD): 7.08; 95% CI: 4.81-9.36), sex hormone-binding globulin (SHBG) levels (MD: −118.61 nmol/L; 95% CI: −174.46 to −62.75), and testosterone levels (MD: 0.48 nmol/L; 95% CI: 0.32-0.64). Conversely, metformin outperformed COCP in reducing fasting insulin levels (MD: −27.12 pmol/L; 95% CI: −40.65 to −13.59) [52]. In a separate systematic review by the same group, the effects of multiple insulin sensitizers were evaluated, including metformin, rosiglitazone, and pioglitazone. The findings indicated that metformin was deemed superior to rosiglitazone in inducing weight loss (MD: −4.39 kg; 95% CI: −7.69 to −1.08 kg), body mass index (BMI) (MD: −0.95 kg/m²; 95% CI: −1.41 to −0.49 kg/m²), and testosterone levels (MD: −0.10 nmol/L; 95% CI: −0.18 to −0.03 nmol/L). However, no significant difference was observed when comparing metformin to pioglitazone [53]. He et al. assessed the efficacy of antioxidant supplementation in PCOS, and this meta-analysis revealed that the fasting blood glucose levels [standardized mean difference (SMD): −0.31, 95% confidence interval (CI): −0.39 to −0.22, p < 0.00001], the homeostatic model assessment of insulin resistance (SMD: −0.68, 95% CI: −0.87 to −0.50], p < 0.00001), and insulin levels (SMD: −0.68, 95% CI: −0.79 to −0.58, p < 0.00001) were significantly lower in patients with PCOS taking antioxidants than those in the placebo group [54].

The medical management of endometriosis uses a multifaceted approach. Progestins, such as dienogest, are often used as first-line treatments, and treatment is mainly aimed at reducing pain and improving fertility outcomes [55]. Medical treatment can greatly improve the quality of life. As this is a long-term approach, drugs must have a good side effect and tolerability profile. In addition to progestins, the levonorgestrel-releasing intrauterine system (LNG-IUS) is also used for management, as synthetic estrogen and progestin-containing preparations have an anti-inflammatory effect on endometrial tissue, thus causing atrophy of the endometrial and ectopic endometrium. This approach also causes amenorrhea, which decreases trans-tubal reflux of endometrial cells. Naturally, this is not suitable for patients trying to conceive; however, cyclical regimens can be tailored as per patient response. Depot medroxyprogesterone acetate is a second-line treatment; again, the mode of administration can be tailored according to adherence and response. In addition, oral GnRH analogs (like Elagolix), with add-back therapy, are another treatment modality that works by inducing a hypoestrogenic state but comes at the cost of inducing a climacteric state, often inducing unpleasant side effects. Administering elagolix was seen to create a significant reduction in dysmenorrhea for patients, as evidenced by Taylor et al. who, through a double-blind randomized six-month phase 3 trial, randomized a sample of 1,689 women with endometriosis to receive dosages of either 150 mg once daily or 400 mg (200 mg twice a day) of elagolix, with a control group of women with endometriosis receiving a placebo. The study ran two randomized controlled trials simultaneously, each with three groups: a higher-dose group, a lower-dose group, and a control group (EM-I and EM-II). At the three-month mark, women in EM-I saw a reduction in their pain by 47% in the lower dose group and 76% in the higher dose group, a 20% reduction in the placebo group (p < 0.001). The EM-II group of women reported a 43%, 72%, and 23% reduction in dysmenorrhea in the lower-dose, higher-dose, and placebo groups, respectively (p < 0.001). However, the women on the higher dose experienced unpleasant climacteric side effects such as hot flashes and lower bone mineral density when compared to baseline, and dyslipidemia, which was not seen in the placebo group, and most women were amenorrheic, although the use of barrier contraception was encouraged as ovulation is not fully suppressed with this regime [56,57].

Supplementation with omega-3 is encouraged as it has been shown to reduce the growth of endometrial tissue and reduce inflammation, as opposed to consuming foods like red meat, which, although rich in omega-3, contributes to inflammation due to its high estradiol and estrone sulfate content [58]. Vitamin D supplementation has also been shown to greatly reduce dysmenorrhea, as seen by Lasco et. al., who reported a negative correlation between pain scores at baseline and vitamin D levels (r = -0.36; p < 0.02). A significant reduction was seen in pain scores when the vitamin D group was compared to the placebo group (p < 0.001). Over an eight-week duration, the largest reduction in pain was reported by women with severe pain at baseline in the vitamin D group (r = 0.76; p = 0.001). Women taking vitamin D did not report using NSAIDs to manage their dysmenorrhea across the eight-week period, as compared to 40% of women in the placebo group taking NSAIDs at least once to manage pain within the same period (p = 0.003) [59]. Khalifa et al., following an RCT on women with endometriosis-related infertility, concluded that gonadotropin-releasing hormone agonists (GnRH) and dienogest, a fourth-generation selective progestin pretreatment, improved ovarian stimulation/number of mature oocytes (6.6 ± 1.3 vs. 6 ± 1.8, p = 0.71), number of transferable embryos (4.5 ± 1.8 vs. 5.1 ± 2.0, p = 0.63), pregnancy rates (22.39% vs. 25.37%, p = 0.69), and clinical pregnancy rates (17.91% vs. 25.37%, p = 0.29) without any significant difference among the groups [52]. Qing et al.'s group of endometriosis patients who underwent surgery after receiving adjuvant GnRH prior to starting ART had a slightly higher pregnancy rate (RR = 1.20, 95% CI = 1.02-1.41; p = 0.03) and a shorter mean time to conception (RR = -1.17, 95% CI = -1.70- -0.64; p < 0.0001) [60].

The most common reasons for diagnosed infertility are tubal disease and ovulatory dysfunction in females and male factors, and the treatment can be tailored according to the cause as medical management is available to be used as an adjuvant therapy. A large percentage of women dealing with infertility in relation to ovulatory dysfunction find its root cause in a PCOS diagnosis [48]. Assuming that the infertility is not caused by the male factor and the female partner does not suffer mechanical concerns that diminish fertility such as pre-existing adhesions and patent fallopian tubes, medical management is a viable first-line option [61]. For PCOS-related infertility, CC as evidenced previously in this paper can be used as a first-line treatment, while aromatase inhibitors like letrozole are used more in an off-label fashion but has a similar functioning to CC, if CC is found to not help, FSH-induced ovulation can be used, using a progressive increasing dose regime as per response. To manage hyperthyroidism caused by Graves' disease, surgical thyroidectomy or anti-thyroid medicines may be recommended. Women should be stably euthyroid before attempting a pregnancy. If increased prolactin is found to be the reason for infertility due to suppression of the hypothalamic-pituitary axis, medical management such as dopamine agonists (cabergoline and bromocriptine) can be used until resolution of hyperprolactinemia and associated oligomenorrhea. Once functional and organic causes of female infertility have been ruled out, the diagnosis of functional hypothalamic amenorrhea can be considered, the treatment of which mainly consists of increasing BMI to a minimum of 18.5, through decreased exercise and a higher caloric intake, with gonadotrophin therapy being evidenced to show improvement in outcomes as well for these patients [62].

Surgical Management of PCOS, Endometriosis, and Infertility

Surgical management of PCOS is not used as a first-line modality but may include bariatric technique in women with BMI above 40, rather than reproductive surgery to improve metabolic outcomes and thus ease symptoms. Multiple reviews have suggested the superiority of metformin and the CC protocol in improving obstetric outcomes as compared to ovarian drilling, which is considered the second-line therapy in the management of PCOS infertility [63]. 

Surgical management in endometriosis aims to facilitate the removal of lesions, with absolute indications for surgery including large endometriomas, adnexal masses with imaging uncertainty, ureteral stenosis causing hydronephrosis, and bowel stenosis [64]. Surgical management of endometriosis consists of various modalities, one of them being excisional procedures once pathological endometrial tissue is identified. If the infiltration by endometrial tissue is limited (AFS classes I and II), a small portion of fibrotic tissue can be left excised without a safety margin, but there exists discourse here. For vaginal cuff or bladder endometriosis, complete excision without a margin is advocated to reduce the risk of recurrence, while excision from the bowel can have a rim of fibrosis left behind as the risk of recurrence does not change pre- or post-operatively. Treatment approaches, however, can vary by the depth of tissue present, i.e., for deep endometriosis (AFS class II), which often requires prior imaging for diagnosis, a conservative excisional approach done with discoid excisions or bowel resection, mainly sigmoid resection alongside anastomosis in cases with large rectal nodules and bowel obstruction [65]. Other forms of bowel surgery and associated outcomes for endometriosis were analyzed in a systematic review by Bendifallah et al. and reported relatively low rates of post-operative complications such as rectovaginal fistulas when performing rectal shaving for endometriosis when compared to disc excision (OR = 0.19; 95% CI (0.10-0.36), p < 0.00001, I^2^ = 33%) and segmental resection (OR = 0.26; 95% CI (0.15-0.44), p < 0.00001, I^2^ = 0%) [66].

For small and surface-level lesions, CO_2_ lasers can be used for vaporization alongside bipolar coagulation; however, efforts to use excision even for superficial lesions should not be discounted, caution should be exercised when using bipolar coagulation with lesions whose depth cannot be assessed, and thus excision becomes the preferred method of treatment here. Cystic ovarian endometriosis, often associated with adhesions (AFS class III/IV), can be managed using transclude-hydro-laparoscopy due to its ability to not add to the burden of postoperative adhesions. This modality is beneficial for small ovarian endometriomas in young patients. Excision here also proves beneficial due to it reducing the rate of recurrence, but successful outcomes are dependent on surgeon skill and can contribute to significant ovarian damage if the surgeon isn’t skilled enough. Superficial destruction of ovarian endometriomas poses a high rate of recurrence and thus is not preferred. Weighing surgical completeness against nerve damage as well as postoperative adhesions (that contribute to infertility, chronic pain, or intestinal obstruction) due to surgery should be evaluated at each step, and preservation of organ function and fertility should be prioritized [65,67].

According to a systematic review by Singh et al., laparoscopy is still considered the gold standard modality in the diagnosis and therapeutic management of endometriosis [67]. They looked at the postoperative outcomes of various surgical treatment modalities used for endometriosis and reported outcomes of laser excision, demonstrating only 11.8% reported they saw no reduction in pain, measured with the visual analog scale; however, 25% reported still being in pain post-surgery and 22.6% reported requiring another surgery. Lesion excision did show a 3.6 cm decrease in score on the visual analog scale. Only 6% of the women studied did not find relief post-pelvic denervation. Adhesiolysis had a high rate of postoperative complications, as seen in this retrospective cohort study by Clark et al., and thus is not a preferred treatment modality. Hysterectomy without ovarian preservation is only considered once all other forms of treatment have been exhausted [68]. 

The surgical approach to treating infertility is mostly focused on managing tubal disease, which does not qualify for medical management, hydrosalpinx can be managed using a salpingectomy before conception or embryo transfer is achieved [69]. In young women with concomitant endometriosis (stage 1/2) and infertility intrauterine insemination can be considered, and IVF for patients with more advanced disease. For women unresponsive to the above-mentioned approaches, endometrial excision and ablation as outlined previously in this paper can be used to increase pregnancy rates [70].

**Figure 3:**
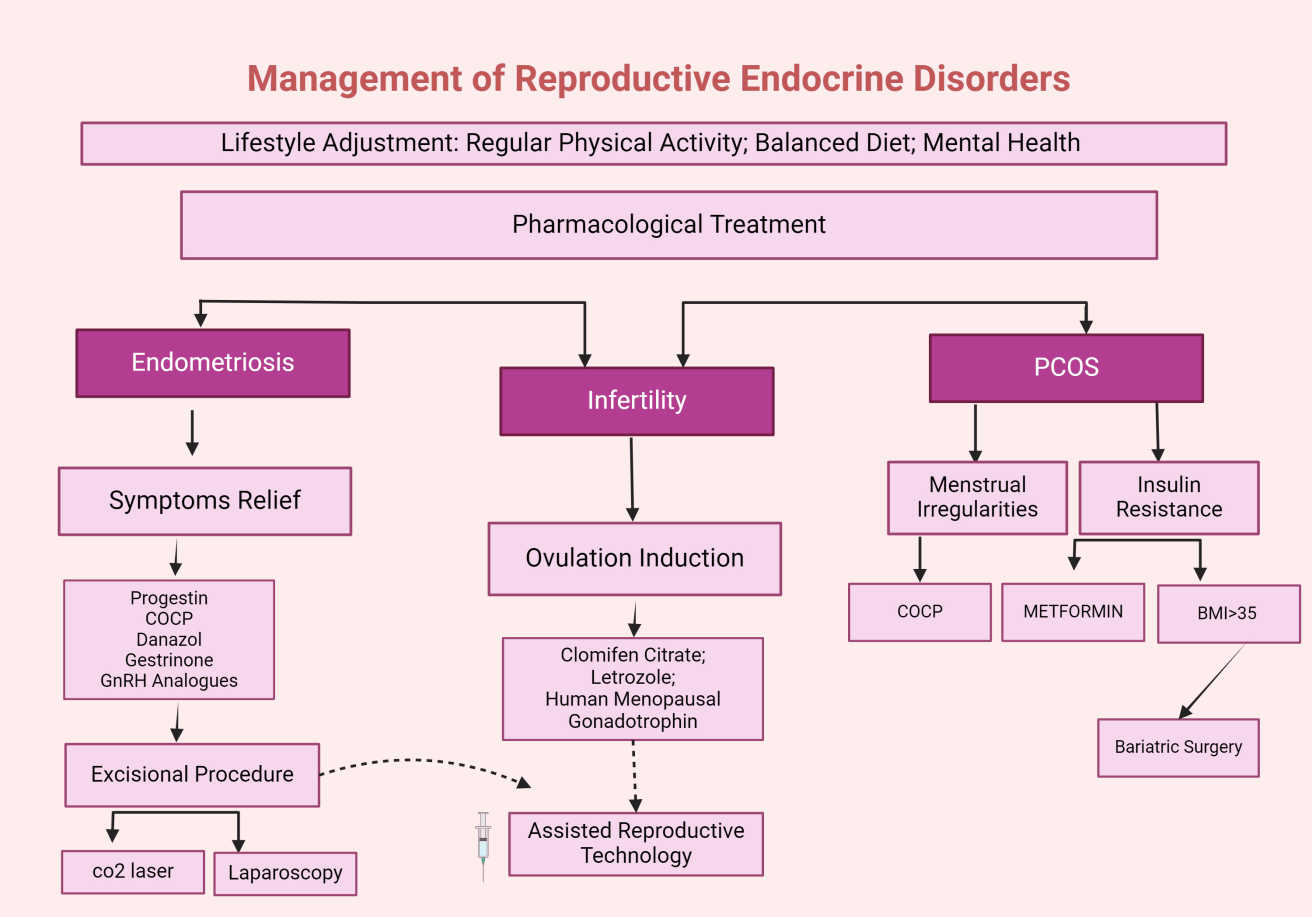
Comprehensive management of reproductive endocrine disorders Created in BioRender (Mendes De Freitas B (2024), BioRender.com/r82m596)

Figure 3 summarizes the management strategies for reproductive endocrine disorders.

## Conclusions

Reproductive endocrine disorders, particularly infertility, PCOS, and endometriosis, continue to pose significant challenges for women’s reproductive health. The complexity of these conditions demands a multidisciplinary approach to ensure optimal care. While lifestyle changes and medical management, such as ovulation induction, provide effective treatment options for many women, surgical interventions may be necessary in more severe cases, especially with endometriosis. Early and accurate diagnosis is essential for improving fertility outcomes and minimizing the impact on a woman's quality of life. Future advancements in diagnostic techniques and treatment strategies will further enhance the ability of healthcare providers to tailor interventions, ensuring better management of these conditions and improved reproductive health.
